# Treatment of Radiation-Induced Brain Necrosis

**DOI:** 10.1155/2021/4793517

**Published:** 2021-12-24

**Authors:** Xiaojing Yang, Hanru Ren, Jie Fu

**Affiliations:** ^1^Department of Radiation Oncology, Shanghai Jiao Tong University Affiliated Sixth People's Hospital, No. 600, Yishan Road, Shanghai 200233, China; ^2^Department of Orthopedics, Shanghai Pudong Hospital, Fudan University, Pudong Medical Center, Shanghai 201300, China

## Abstract

Radiation-induced brain necrosis (RBN) is a serious complication of intracranial as well as skull base tumors after radiotherapy. In the past, due to the lack of effective treatment, radiation brain necrosis was considered to be progressive and irreversible. With better understanding in histopathology and neuroimaging, the occurrence and development of RBN have been gradually clarified, and new treatment methods are constantly emerging. In recent years, some scholars have tried to treat RBN with bevacizumab, nerve growth factor, and gangliosides and have achieved similar results. Some cases of brain necrosis can be repairable and reversible. We aimed to summarize the incidence, pathogenesis, and treatment of RBN.

## 1. Introduction

Radiation therapy is regarded as an important therapy to treat brain tumors, and its efficacy has also been confirmed [1]. However, radiation therapy involves the risk of nerve damage, including focal cerebral necrosis, neurocognitive dysfunction, cerebrovascular disease, myelopathy, and brachial plexus neuropathy [2, 3]. The occurrence and development of radiation-induced brain necrosis (RBN) depend on the total radiation dose, the fraction size, and the volume of the brain. In general, the higher the total radiation dose is, the larger the split dose is, the larger the brain volume is, and the higher the incidence of RBN [4]. The higher the total radiation dose, the easier will be the early occurrence of RBN. This tumor type manifests as headache, insanity, dizziness, memory loss, personality changes, and seizures [5]. These symptoms severely affect the quality of life of patients. In this review, we aimed to summarize the incidence of RBN, its pathogenesis, diagnosis, and treatment plans and strategies, as well as the prognostic indicators and how to prevent these.

## 2. Epidemiology

RBN is a serious complication of intracranial and skull base tumors after radiotherapy. Previous studies included data on the frequency of RBN after irradiation of nasopharyngeal carcinoma (NPC), glioma, brain metastases, and intracranial arteriovenous malformations and are summarized in Table 1 [6–15]. Ruben et al. [15] have reported that adjuvant chemotherapy after radiotherapy increased the incidence of RBN by a factor of five. Also, the study reported that the incidence of RBN in patients with survival of more than 1 year after conventional irradiation for malignant gliomas ranged from 10% to 15% [15]. The incidence of temporal lobe necrosis in patients with nasopharyngeal carcinoma within 9 months to 16 years of conventional radiotherapy (dose below 6000 cGy) ranged from 1.6% to 22.0% [16]. Lee et al. [17] have reported the incidence of temporal lobe necrosis within 10 years after receiving conventional segmental radiation therapy for nasopharyngeal carcinoma, which was about 5%. The actual incidence of temporal lobe injuries after routine radiotherapy of NPC remained as high as 34.9% according to the Sun Yat-sen University Cancer Center. With widespread application of intensity modulated radiation therapy (IMRT) whose a fraction size < 2 Gy, the incidence of RBN has been decreased [18]. This is mainly due to better spare of IMRT of normal tissues surrounding the tumors than conventional radiotherapy techniques. Symptomatic focal cerebral necrosis occurs in 2% to 5% of patients with brain metastases after stereotactic radiosurgery (SRS) [14]. Intracranial arteriovenous malformations require surgical intervention in 4% to 5% of patients after receiving SRS due to symptomatic cystic changes or focal necrosis [19]. Neuroprotective therapies for radiation-induced brain injury remains limited [20].

## 3. Pathophysiology

RBN begins with radiation-induced vascular injury, which occurs within the first 24 hours after radiation, and followed by parenchymal brain injury [21]. Ionizing radiation induces reactive oxygen species in tumor cells, resulting in single- and double-stranded DNA damage. The DNA repair pathways are then subsequently activated, resulting in cell cycle arrest and irreversible damaged DNA apoptosis [22]. Radiation also interacts with cytoplasmic membrane, which in turn destroys endothelial cells and causes ceramide-induced apoptosis [23]. This triggers a series of events that lead to cell swelling and necrosis, production of more reactive oxygen species, and subsequent transmission of inflammatory responses involving cytokines and chemokines. The formation of fibrin-platelet thrombus and fibrinoid necrosis resulted in blood-brain barrier disruption and brain edema [24, 25].

**Figure 1:**
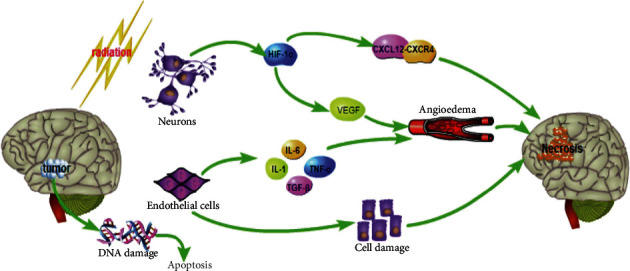
Relevant mechanisms involved in the process of RBN occurrence.

According to the pathology of RBN, the main features of it included white matter necrosis, fibroid-like necrosis, hyaline degeneration of blood vessel walls, and capillary dilatation. So, RBN is histologically defined as cellulosic vascular necrosis and with persistent inflammation. With the popularization of MRI technique for brain tumor and nasopharyngeal carcinoma after radiotherapy, more and more RBN cases have been reported and studied. Based on the published literature reports, the occurrence of RBN remains a very complex and multi-factor interaction process. There are currently three accepted hypotheses for the occurrence and development of RBN: (1) vascular injury, (2) glial cell and white matter injury, and (3) inflammatory response and abnormal cytokine expression [26–29]. The relevant mechanisms involved are shown in Figure 1. Among these, vascular injury is recognized hypotheses for the occurrence of RBN in the acute phase, which is earlier than the subacute demyelinating reaction and the astrocyte and microglia reactive response.

### 3.1. Vascular Injury

The destruction of astrocyte function after radiation may lead to an imbalance in the overall brain homeostasis, leading to functional defects [30]. Neuronal damage or radiation-induced activation of the complement cascade leads to destructive oligodendrocyte activation [31]. Studies have determined the destructive effects of radiation-induced oligodendrocyte activation in the irradiated brain [32]. RBN may be a consequence of damage to oligodendrocyte progenitor cells and vascular endothelial cells. Vascular structure changes play a vital role in the central nervous system toxicity caused by radiation. The earliest observations on histology included vascular endothelial basement membrane bulge, swollen nuclei, and cytoplasmic vacuolation, leading to changes in the capillary permeability, and causing angioedema finally [33]. Vascular endothelial cell damage occurs during the chronic injury phase. The pathological manifestations included endothelial injury, capillary dilatation, and vasodilation, leading to increased permeability at the site of vascular injury, and finally the formation of vascular edema [34]. This process occurs within weeks to months after the initiation of radiotherapy. Typical manifestations include partial or complete blockage of blood vessels with thrombus, subsequent abnormal proliferation of endothelial cells, thickening of the basement membrane, and replacement of the lumen with collagen [35]. However, the mechanisms that regulate endothelial cell proliferation, collagen production, and basement membrane thickening still remained unclear. In summary, progressive vascular changes include wall thickening, thrombosis, infarction, and necrosis due to hyaline degeneration. The above phenomenon proves that vascular injury plays an important role in brain toxicity caused by radiation, but this hypothesis has not been neither confirmed nor universally accepted.

### 3.2. Glial and White Matter Damage

In addition to the damage of endothelial cells, radiation also damages astrocytes, oligodendrocytes, and neural progenitor cells [36, 37]. This results in inflammatory response and formation of necrotic tumor fragments that are difficult to remove, exacerbating capillary permeability defects and promoting demyelination. These changes have been considered difficult to separate from tumor progression [21]. The irreversible delayed period of RBN involves a series of characteristics, ranging from focal radiation necrosis to diffuse brain atrophic white matter encephalopathy. Brain damage caused by radiation is mainly found in the white matter [21].

Recently, the role of vascular endothelial growth factor (VEGF) and hypoxia-inducible factor-1*α* (HIF-1*α*) in the pathogenesis of brain radiation necrosis has become more apparent. HIF-1*α* is an inversely activates of VEGF, while its upregulation increases VEGF production by astrocytes, leading to angiogenesis [38]. However, the blood vessels the resulted from this response are brittle and leaky, which causes edema around the lesion, and this is the characteristic of acute phase of brain necrosis. Increased VEGF was found in the brain necrosis areas of animal models [39]. HIF-1*α* is also considered as an important modulator of chemokine axis mediator CXCL12-CXCR4, and its inhibitory effect reduce the development of brain radiation necrosis in animal models [40].

Another typical pathological change in RBN is demyelination, in which the O-2A cells acts as precursor cells for type II oligodendrocytes, and is quite sensitive to radiation. O-2A cells not only produce mature oligodendrocytes that are necessary for myelin formation but also differentiate into type II stellate cells and participate in the maintenance of unique electrophysiological properties of Lange nodules. Radiation causes loss of proliferation of these cells in the brain and spinal cord of adult rats [41]. It has been speculated that the radiation-induced deletion of O-2A cells led to abnormal proliferation of oligodendrocytes, eventually causing demyelinating changes [42]. In addition to killing O-2A cells, radiation also directly kills oligodendrocytes. In vitro studies revealed that oligodendrocytes, and not O-2A stem cells, undergo apoptosis after irradiation [43]. Subsequent in vivo studies have reconfirmed that oligodendrocytes undergo apoptosis after spinal cord irradiation in rats [44]. Studies have shown that cytokines such as TNF-*α* induces oligodendrocyte death, and so it is speculated that in addition to direct killing, release of radiation-induced TNF-*α* also increased the toxicity of oligodendrocytes [45]. The kinetics of oligodendrocyte loss remains unstable, but eventually led to necrosis [46].

Radiation not only affects blood vessels and O-2A cells but also microglia, astrocytes, neurons, and neural stem cells. Although the neurons are relatively resistant to radiation, a certain number of cells are still lost after irradiation. Cell loss occurs mainly in white matter, and this is why the brain volume shrinks after radiation brain damage. Other confirmed metabolic change after brain irradiation is decreased glycolysis, and this is exactly associated with the reduction of glucose and oxygen utilization during PET imaging of patients with radiation brain necrosis [47]. In vivo experiments in animals have confirmed that DNA double-strand breaks of neurons and stellate cells increase proportionally with increasing dose. Low dose of 2 Gy irradiation mainly induces typical apoptosis of neurons, while high-dose 32 Gy irradiation has little or no apoptosis. Radiation-induced neuronal apoptosis occurrs 4 to 8 hours after irradiation and peaks at 12 hours [48]. In vivo experiments in adult rats also confirmed that radiation can induce neuronal apoptosis, but the apoptosis is limited to the epithelium. Cells in this area are present in a mitotic active phase producing glial and neuronal precursor cells [49].

### 3.3. Inflammatory Response and Abnormal Cytokine Expression

RBN tissue showed the coexistence of new and old inflammatory reactions under the microscope, suggesting that the occurrence and development of the mechanism of RBN can be divided into two stages: tissue damage and inflammatory response [29]. The illuminated endothelial cells and inflammatory cells secrete different cytokines, such as VEGF, tumors necrosis factor (TNF)-*α*, interleukin (IL)-1*α*, IL-6, and transforming growth factor (TGF)-*β* [50, 51]. This chronic inflammation, which is different from cytokines, might play a role in the development of RBN due to excessive production of proinflammatory cytokines, which is the pathophysiological mechanism of many neurodegenerative diseases [52], but how the abnormal expression of cytokines and the inflammatory response eventually led to brain necrosis needs further study.

Radiation can directly damages the glial cells and endothelial cells of the brain, leading to hyalinization and demyelination of blood vessels, followed by inflammation, ischemia, and delayed radiation necrosis. Many studies have suggested that postradiation neuroinflammation is linked to brain damage and cognitive impairment [53–55]. Radiation-induced neuroinflammation involves a crossnetwork of multiple pro- and anti-inflammatory cytokines. Microglia plays a major role in neuroinflammation [56, 57]. Radioactive rays activate microglia by altering their cell morphology and function [58]. There are two different types of microglial cells after activation: the classic M1 and the alternative M2 activation types. M1 microglia might become phagocytic cells and may synthesize proinflammatory molecules such as IL-1b, TNF-*α*, IL-6, and superoxide radicals and nitric oxide (NO), which in turn help in clearing the infected and repair tissues [59]. On the other hand, M2 activation types are related to anti-inflammatory cytokines such as IL-10, insulin growth factor-1 (IGF-1), and neurotrophic factors [59], limiting neuronal damage and promoting healing [60]. However, although microglia activation plays a vital role in brain pathology, the specific mechanism of microglia activation and polarization, the downstream molecular cascade and how to regulate this process warrants further study [61]. Studies by Chen et al. [62] showed that two proinflammatory factors TGF-*β*1 and TNF-*α* are associated with radiation-induced damage, which are significantly increased shortly after radiotherapy and rapidly decreased one month after radiotherapy. On the other hand, the anti-inflammatory cytokine IL-10 is elevated during and after radiotherapy. The activation of immune cells plays a key role in the blood-brain barrier (BBB). The activated brain immune cells upregulate the expression of proinflammatory factors and chemokines and activate matrix metalloproteinases (MMPs) to destroy the integrity of the BBB and recruit peripheral immune cells to the injured area, leading to secondary BBB damage [63]. Changes in these inflammatory cytokines further indicate that they are affected by radiation and might play an important role during the process of RBN.

## 4. Mechanism

Stone and DeAngelis [64] reported that radiation might affect cognitive deficits, hippocampal nerve damage, and cerebellar dysfunction. Based on the time after radiotherapy, the side effects caused by radiation were divided into three phases: acute response period (several days to weeks), delay period (1-6 months), and delay period (>6 months) [65]. Previous research has focused on late-stage radiation-induced brain injury, including functional and structural defects [8]. According to recent research, radiation-induced brain damage might probably occur early [66] and lead to future cognitive dysfunction [67].

In radiation-induced brain injury, BBB is destroyed, causing systemic immune and inflammatory cells to enter the brain and promote the pathway of neuroinflammation [68]. Neuroglia induced by microglia might be considered as the main checkpoints and involve mediation of many cellular interactions that lead to dysfunction after whole brain radiation [69, 70]. In vitro studies have revealed that microglial cells are activated after radiation and subsequently lead to increased expression of various proinflammatory genes, including TGF-*β*1, IL-10, IL-6, TNF-*α*, and cyclooxygenase (COX)-2 [71]. Previous studies have found that radiation-activated microglia-mediated neuroinflammation plays a key role in the development of radiation-induced brain injury [72]. Multiple studies have shown that cytokines are directly or indirectly involved in the development of radiation damage [73, 74]. TGF-*β*1 can regulate immune inflammatory response as a two-way regulator of proinflammatory or anti-inflammatory response [75]. TNF-*α* has become one of the most critical profibrotic cytokines, and IL-10 inhibits inflammatory response and reduces macrophage activity [74].

After the exposure of the brain to radiation, an inflammatory response occurs [76]. Within hours of radiation, the microglia are activated, the shape of the cells change, and the damaged nerve transcription factors are activated and proinflammatory mediators are produced [77], leading to damage of the central nervous system [78]. It has also been reported that radiation depletes neural progenitor cells in the subgranular zone of the hippocampal dentate gyrus and inhibits neurogenesis [79]. Therefore, inhibition of destructive inflammation and promotion of neurogenesis might limit radiation-induced brain damage.

Voltage-gated Kv1.3 potassium ion channels may play an important role in different cell types (microglia, T cells, dendritic cells, and NPC cells) involved in radiation-induced central nervous system damage. Kv1.3 is upregulated during microglial activation [80], and microglial-mediated neuronal damage requires Kv1.3 channel activation [81]. Kv1.3 can regulate the immune functions which is important in the monitoring and killing of cancer cells [82]. Targeting Kv1.3 channel with a selective blocker can reduce RBN by targeting key cells involved in it, subsequently promoting neurogenesis. We can guess that Kv1.3 might also be a factor for RBN. Studies have shown that Stichodactyla helianthus- (ShK-) 170 (a Kv1.3 selective peptide inhibitor) inhibits microglial activation as well as the production of proinflammatory factors and promotes nerve repair to improve radiation-induced brain damage [1]. Gene silencing with Kv1.3-specific siRNA and pharmacological blockade with ShK-170 in radiation microglia have significantly reduced the production of proinflammatory factors. ShK-170 also effectively reduced the activation of microglia and the production of proinflammatory factors after head irradiation. Although radiation directly caused neuronal apoptosis, radiation-activated microglia aggravated this damage by producing proinflammatory factors. ShK-170 significantly reduced radiation-activated microglial-mediated neurotoxicity, but failed to protect the neurons from the direct neurotoxic effects of recombinant IL-6 and TNF-*α*. ShK-170 therapy also reduced neuronal damage in the hippocampal cortex and CA3 area, enhanced neural stem cell proliferation, and inhibited microglial-mediated neurotoxicity. These results suggested that ShK-170 limits the radiation-induced brain damage by targeting two key processes: inhibition of microglia-mediated neuroinflammation, thereby protecting the neurons from proinflammatory factor-mediated toxicity, and promotion of the occurrence and repair of the nerves.

## 5. Diagnosis

Patients diagnosed with radiation necrosis must have a history of head and neck radiotherapy; RBN can appear after radiotherapy for benign and malignant tumors, and it is more common in malignant tumors, which may be related to the high radiation dose; RBN usually occurs half a year to a year after radiotherapy. Patients treated with multiple radiotherapy methods are more susceptible to RBN. Although pathological biopsy is the gold standard, due to the invasiveness of biopsy and the large sampling error, in general, RBN needs to be combined with the patients' medical history, symptoms and imaging examinations to confirm the diagnosis [83, 84].

## 6. Treatment

**Figure 2:**
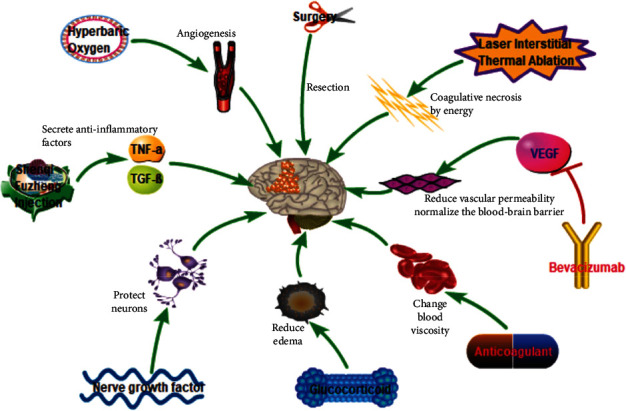
Summary of various treatment methods of RBN and related mechanisms involved.

The symptoms, disease status, and development of suspected lesions on diagnostic imaging are considered as important factors when dealing with RBN. It is also important and necessary to involve patients and family members in the decision-making process and to understand the natural course of RBN, available treatments, and possible outcomes [85]. For small, asymptomatic lesions, an observational wait strategy, with continuous clinical follow-up can be adopted, wherein this is supplemented with continuous diagnostic imaging. Close imaging follow-up is usually recommended during the beginning (every 6-8 weeks) of the strategy at short intervals until the occurrence of the lesions. If the size of the lesion is stable or reduced, then the frequency of follow-up can be reduced according to the specific situation [86]. For asymptomatic brain necrosis, the treatment strategy usually involves follow-up observation while for symptomatic brain necrosis, and the classic treatment involves relieving of the symptoms by surgery, glucocorticoids, or anticoagulants. Some scholars have also tried to treat RBN with hyperbaric oxygen and high-dose vitamins. Recently, with the understanding of the pathophysiology of RBN and the development of new drugs, some scholars have attempted to use new interventions for treat RBN (such as bevacizumab, nerve growth factor and gangliosides). The treatment results in recent years are summarized in Table 2 [5, 62, 87–104]. We summarize various treatment methods and related mechanisms involved in Figure 2.

### 6.1. Glucocorticoid

The most common treatment for RBN involves the use of glucocorticoids to control necrosis-related edema. Dexamethasone usually assists in quickly relieving the clinical symptoms caused by focal necrosis. 500 mg of dexamethasone was dissolved in 250 ml of 0.9% saline solution, and the dose was gradually reduced during the course of use until the end of 14 days of treatment [105]. Some cases after long-term application of corticosteroids showed a partial remission in imaging, but this remission remained temporary in most of the cases, and the patient eventually develops hormonal dependence. It is well known that long-term application of glucocorticoids can lead to secondary chronic complications. For example, increased number of infections, stomach pain, hyperglycemia, cataracts, osteoporosis, peptic ulcer disease, liver damage, and bone aseptic necrosis [106, 107]. There are several case series that suggested intravenous administration of glucocorticoida might pose a serious risk of liver disease, and the degree and outcome of severe liver damage are dose-dependent [108]. Compared with high-dose steroids, the study by Zhuo et al. showed that low-dose steroids are safer and more effective, and that the overall incidence of adverse events remained low. Treatment-related infections are associated with high-dose steroids rather than low-dose steroids, which might induce immunosuppression [109].

### 6.2. Anticoagulant

RBN might be mainly caused by ischemia due to vascular injury, and so some scholars have used anticoagulant therapy to prevent RBN progression. It has been reported that the clinical symptoms of cerebral necrosis are partially relieved after anticoagulation with heparin and warfarin [90]. Before using anticoagulants, the potential risk of bleeding that occurs later should be considered and also the advantages and disadvantages before using them should be weighed. In addition, published studies on the effectiveness of anticoagulation therapy have included only a small number of patients, and large randomized controlled trials have not confirmed the benefits of anticoagulation therapy. Pentoxifylline is a methylxanthine derivative that changes blood viscosity and has been shown to reduce the diffusion of carbon monoxide into the lungs of patients with lung cancer or breast cancer undergoing radiation therapy [110]. Pentoxifylline is proved to reduce skin changes, fibrosis, and soft tissue necrosis caused by postoperative radiotherapy in patients with squamous cell carcinoma of the head and neck. Also, pentoxifylline reduced brain radiation damage to varying degrees [90]. Clinical trials on the use of pentoxifylline and vitamin E to prevent RBN are expected to report their findings in the near future [92].

### 6.3. Bevacizumab

Bevacizumab is a humanized monoclonal antibody that blocks VEGF. Studies have revealed that bevacizumab, whether used alone or in combination with other chemotherapeutics, has a therapeutic effect on a variety of solid tumors. Also, it has been confirmed that bevacizumab can reduce vascular permeability and normalize the blood-brain barrier [111]. Several animal models of RBN increased the expression of VEGF, leading to further deterioration of blood-brain barrier function and cerebral edema [50, 51]. Preventing VEGF from reaching the capillaries is regarded as a reasonable strategy for treating RBN, with the goal to reduce the entry of plasma and water into the extracellular space. Bevacizumab counteracts the effects of VEGF on RBN and reduces the use of steroids. Intra-arterial administration of bevacizumab has successfully treated RBN and continued to respond for 8.5 months after administration. Two retrospective studies have reported on the experience of bevacizumab for RBN. In 14 RBN patients, the clinical symptoms in all cases were alleviated to some extent after bevacizumab treatment. MRI showed that the lesions were partially reduced. But in one case, the enhanced lesions revealed by MRI after bevacizumab treatment has almost disappeared [112]. This suggested that the process of RBN might be reversed. Some scholars have designed a prospective, placebo-controlled, double-blind clinical trial [113]. A total of 14 patients were randomly divided into the saline control group and bevacizumab group, in which bevacizumab was intravenously administered at a dose of 7.5 mg/kg, with an interval of 3 weeks, and was repeated twice. The assessment was performed 3 weeks after second administration of bevacizumab, including MRI objective assessment of the reduction of necrotic lesions and a subjective assessment of clinical symptoms. For effective and no serious complications, the original treatment was continued for 2 cycles. The results of the first evaluation showed that the clinical symptoms of all patients receiving bevacizumab were alleviated to varying degrees, and the volume of necrotic lesions was reduced by MRI. The placebo group showed no response to objective and subjective indicators. After a median follow-up of 10 months for patients receiving bevacizumab, MRI findings revealed progression of necrotic lesion in only 2 cases. Of these, 6 had adverse events, including one pulmonary embolism and one sagittal sinus thrombosis. Therefore, the safety of bevacizumab deserves further verification by large-scale randomized controlled trials (RCTs).

### 6.4. Nerve Growth Factor

**Figure 3:**
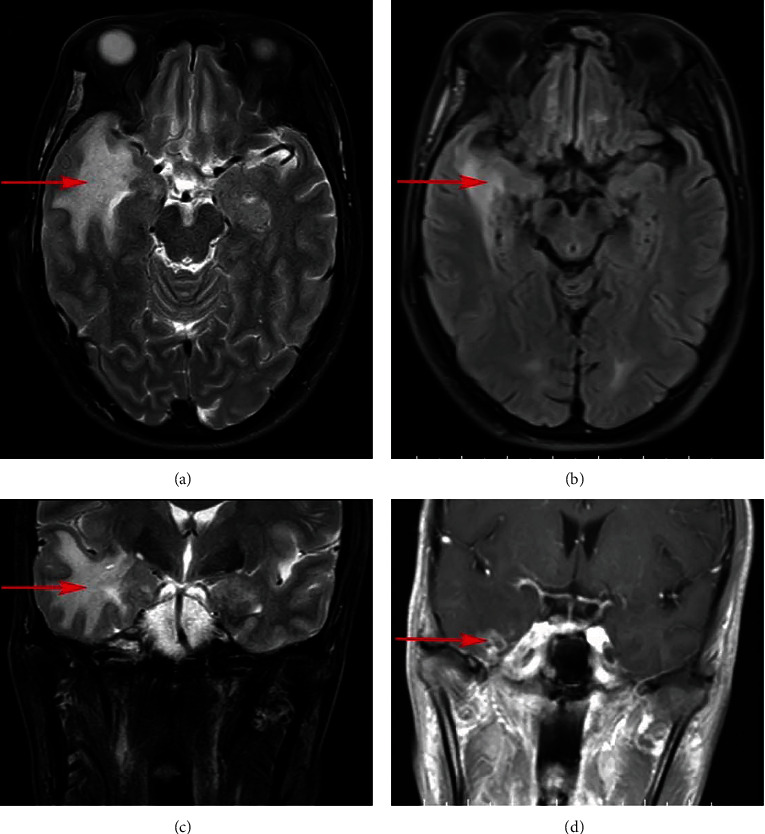
The rat nerve growth factor was used to treat a patient with radiation-induced brain necrosis after radiation treatment of nasopharyngeal carcinoma. (a) (axial plane) and (c) (coronal plane) are MRI images before treatment, and (b) (axial plane) and (d) (coronal plane) are MRI images after treatment (red arrows indicate cerebral necrosis lesions).

Nerve growth factor (NGF) demonstrated obvious protective effects on the central and peripheral nervous system, wherein it prevents neurons from apoptosis and degradation, and promotes the repair and regeneration of injured neurons. Radiation damage of oligodendrocytes and neurons showed association with cerebral necrosis and concluded that nerve growth factor might have a therapeutic effect on RBN. A recent phase II study is aimed at outlining the use of NGF in the treatment of brain radiation necrosis [5]. An article reported a case in which NGF has successfully reversed RBN. One patient with nasopharyngeal carcinoma demonstrated bilateral temporal lobe necrosis after radiotherapy. The rat NGF was intramuscularly injected at a dose of 18 *μ*g for two consecutive months. The MRI was reviewed 3 months after and showed complete repair of bilateral temporal lobe necrosis [114]. The author then conducted a prospective, randomized controlled phase II clinical study to analyze the effectiveness of NGF for the treatment of temporal lobe necrosis. The results showed that NGF can reverse the temporal lobe necrosis caused by radiotherapy of nasopharyngeal carcinoma with minimal toxicity [5]. The rat NGF was used by our team to treat a patient with radiation-induced brain necrosis after radiation treatment of NPC according to the above plan, achieving good results. The MRI images of the patients before and after treatment are presented in Figure 3. Although the future remains promising, NGF is currently not considered as the standard treatment, and so further research is needed to verify its safety and effectiveness. Treatment of RBN with NGF must first rule out tumor recurrence or metastasis, and due to its function, it can promote the growth of nerve cells and might also promote the growth of tumor stem cells. RBNs that occur after radiotherapy for neurologically derived tumors require careful use of NGFs. Because of malignant brain tumors, it is difficult to undergo radical resection. Surgical tumors or tumor recurrences often coexist with RBN after radiotherapy. Currently, it is difficult to clearly identify tumors or necrosis in all imaging studies. When stimulated by NGF, brain tumor cells will grow faster, and the original symptoms will be worsened.

### 6.5. Shenqi Fuzheng Injection (SFI)

Preclinical studies have revealed that anti-inflammatory drugs might improve radiation-induced cognitive impairment in patients with brain ionizing radiation [115, 116]. Early use of anti-inflammatory drugs might be beneficial in limiting and improving cognitive impairment caused by radiation [115, 116]. SFI is a Chinese herbal medicine, and previous studies have shown that it can relieve radiation pneumonitis and cause changes in the levels of TNF-*α* and TGF-*β* at different stages of treatment (i.e., before, during, and after treatment) [117]. By examining the inflammatory factors and BBB integrity in skull-irradiated mice, SFI treatment revealed alleviation of radiation-induced inflammatory damage [118]. Chen et al. have used SFI to treat lung cancer patients with brain metastases and found that SFI might promote the production of anti-inflammatory cytokines, which might subsequently control radiation-induced neuroinflammation and reduce RBN [62]. SFI can increase the activity of superoxide dismutase (SOD) in brain tissue, scavenging free radicals, and weaken lipid peroxidation. SFI can improve the survival rate of brain cells by repairing tissue oxidative stress damage caused by radiation [119].

### 6.6. Surgery

For patients with RBN who are associated with poor response after conservative treatment or need urgent treatment, surgery can be used to remove necrotic lesions. It has the ability to provide tissue diagnosis and research samples rule out the situations as biopsy might miss a tumor. However, there is sufficient evidence to suggest that surgical resection is not necessary, as the symptoms in some cases will resolve on their own after the use of glucocorticoids. Some necrotic lesions are located in areas that cannot be removed by surgery. Even if some necrotic lesions have been surgically removed, the normal brain tissues around the necrotic lesions continue to cause necrosis, leading to continuous progression of symptoms. While some necrotic lesions are diffuse, there is no obvious boundary for surgical resection. In addition, the complications of brain surgery itself cannot be ignored, and there are no reports of survival benefit from surgery when compared to conservative treatment [15].

### 6.7. Hyperbaric Oxygen Therapy (HBOT)

The reason for using HBOT to treat brain radiation necrosis is that the increasing oxygen concentration stimulates angiogenesis, restores blood supply to necrotic lesions, and promotes healing. Patients receive HBOT in a 100% oxygen chamber for up to 5 times per week. This cycle can be repeated up to 40 times. Results showed that 20 HBOT treatments a week after SRS can reduce brain radiation necrosis from 20% to 11% [93]. However, the evidence is limited to case reports and no RCT articles have been published till date.

### 6.8. Laser Interstitial Thermal Ablation (LITT)

LITT relies on the transmission of laser electromagnetic radiation to the target tissue, which absorbs photons and releases thermal energy. This heat is then redistributed through convection and conduction, causing coagulative necrosis of the lesion [120]. LITT might be a promising treatment for lesions that are difficult to remove by surgery. Currently, the use of LITT in brain radiation necrosis is limited, but showed significant improvement in the clinical symptoms of reported cases [104]. To date, there are no RCTs that published LITT for brain radiation necrosis.

## 7. Future Opportunities

ShK-186, a stable analogue of ShK-170, has completed preclinical pharmacokinetic and toxicity studies and has been evaluated in a phase I clinical trial in humans [121, 122]. ShK-186 and clofazimine [123] are drugs with Kv1.3 blocking activity, effectively limiting its ability to damage the nervous system after radiation treatment of brain, head, and neck cancer. Kv1.3 blockers might have advantages over corticosteroids. They are also considered as effective inhibitors of neuroinflammation and brain edema after radiotherapy, but they have disadvantages of inhibiting neurogenesis. Compared with other drugs such as indomethacin and minocycline, Kv1.3 blocker has the advantages of inhibiting microglial activation and having neuroprotective effects [124]. Tetrahydrocurcumin (THC) has antioxidant and anti-inflammatory effects. THC effectively reduces brain edema and repairs the damage of BBB by increasing the activity of SOD [125]. Long-term administration of AL002c (an anti-human TREM2 agonist mAb) alleviated the inflammatory response in mouse microglia. The AL002c variant proved to be safe in the first human phase I clinical trial. AL002 is also expected to become a therapeutic drug for RBN [126].

## 8. Prognostic Factor

The risk of brain radiation necrosis varies with tumor location, histology, and genotype. Based on the imaging evidence, 5747 lesions were analyzed, and 15% of these were brain radiation necrotic lesions. These lesions showed statistical significance between brain radiation necrosis and metastatic lesions of the kidney and non-small-cell lung adenocarcinoma [127]. HER2 amplification, BRAF V600 + mutation status, and ALK rearrangement showed significant association with brain radiation necrosis. O6-methylguanine-DNA methyltransferase (MGMT) is an enzyme that inhibits apoptosis. Methylation of MGMT promoter sequence silences its expression and eventually results in cell death. Methylation of MGMT promoter and mutations in isocitrate dehydrogenase 1 (IDH1) predicted false progression in patients with RBN [128].

Recent whole-genome studies revealed that the risk of radiation necrosis of the temporal lobe brain with different single nucleotide polymorphisms (SNPs) remained different in glioblastoma cell line U87 treated with X-rays and H_2_O_2_. SNP showed the greatest risk of affecting CEP128, which maintains normal ciliary function and might protect against radiation damage [129]. Induction of A > G changes in the CEP128 promoter produced a variant that impaired its promoter activity and led to the knockdown of CEP128. This led to higher apoptosis and cell death in the U87 cell line and showed association with the risk of temporal lobe radiation damage. This is the first study that involves radiation injury sensitivity gene (Cep128) and provides new insights into the underlying mechanisms of radiation-induced brain injury.

Albert et al. [130] believed that over time, radiation-induced early brain damage may form long-term structural changes, leading to permanent cognitive dysfunction. It is therefore necessary to detect early radiation-induced brain damage in patients before the occurrence of any serious and irreversible damage. Recognition of sensitive neuroimaging biomarkers of early radiation-induced brain damage might help to clinically diagnose and minimize brain damage. In addition, due to the complex effects of radiation-induced brain injury over time [131], research on patients' brain injury at different periods is also considered very meaningful for clinical treatment.

Diffusion tensor imaging (DTI) is the only noninvasive technique that can be used to study the microstructure of human white matter (WM). It has been used to detect WM abnormalities in patients with nasopharyngeal carcinoma after radiotherapy [132]. Several DTI studies have reported that WM changes mainly occur in the temporal lobe, parietal lobe, and cerebellum of NPC patients after radiotherapy [133, 134]. The temporal lobes are susceptible to radiation as they are very close to the clinical target volume [67]. Therefore, patients might have radiation-induced temporal lobe changes after radiation therapy [135]. Leng et al. [133] have found that fractional anisotropy (FA) in the right temporal lobe of nasopharyngeal carcinoma patients after radiotherapy showed significant reduction. Xiong et al. [136] have calculated DTI indicators of some regions of interests (ROIs) in the temporal lobe of nasopharyngeal carcinoma patients and found that WM was affected immediately after RT, but was recovered after 1 year. However, previous DTI studies focused on specific brain regions or WM regions in nasopharyngeal carcinoma patients after radiotherapy, and these studies provided information only on the integrity of the brain WM in isolated or predefined WM regions [136]. Hyponatremia was identified as a potential predictor for the progression of patients with RBN [137]. Cai et al. [138] have developed a Norfolk study to evaluate the relationship between radiotherapy and cerebral necrosis and found four important predictors: hypertension, statin therapy, serum high-density lipoprotein levels, and the interval between radiotherapy and cerebral necrosis time.

The angiogenic factors vascular endothelial growth factor (VEGF) and angiopoietin can be used as biomarkers of RBN. It is also possible to predict the progression and survival results of radiation damage by measuring the chromosome damage caused by radiation [139].

## 9. Prevention

Although literature reports showed that bevacizumab and NGF as effective treatment strategies for the treatment of RBN are considered as the most effective and cost-effective method for prevention, the main clinical practice involves the use of advanced radiotherapy techniques, such as IMRT, to reduce the volume of normal brain tissue that is exposed to high doses of radiation, or to reduce the maximum dose of brain tissue to avoid or reduce the incidence of RBN. Sun Yat-sen University Cancer Center has retrospectively analyzed 500 patients with nasopharyngeal carcinoma who were followed up for >6 months (305 patients in the IMRT group and 195 patients in the conventional radiotherapy group). The actual incidence of temporal lobe necrosis within 5 years was 16.0% in the IMRT group, while that in the conventional radiotherapy group was 34.9%. Further analysis showed that the protective effects of IMRT on temporal lobe when compared with conventional radiotherapy were mainly reflected in patients with stages T1-3. The incidence of temporal lobe necrosis in patients with T4 stage IMRT and conventional radiotherapy remained similar [140].

Another measure that might reduce RBN is stem cell-based applications. The previous section described that radiation can lead to the loss of O-2A cell proliferation ability and eventually cause demyelination. Studies have shown that transplantation of purified O-2A cells into demyelinated regions can stimulate myelination [141]. Groves et al. [142] have transplanted pluripotent embryonic stem cell-derived precursor cells into a rat model of human demyelinating disease. These precursor cells interact with host neurons to fully myelinate axons in the brain and spinal cord. Ijichi et al. [143] have conducted another animal experiment, and the results showed that transplantation of cells expressing platelet-derived growth factor can increase the number of O-2A cells without affecting the proliferation potential or differentiation capacity of O-2A cells in vitro. There also have some clinical reports on the applications of stem cells to prevent RBN [144, 145]. In preclinical research, people use various stem cell therapies to restore the neurogenic niche [146].

## 10. Conclusions

Although the incidence of RBN remained low, it affected the quality of life of patients. Close monitoring of functional imaging of the brain after radiotherapy remained essential. There is currently no unified treatment plan for radiation necrosis, but alternative treatments are increasing, and certain effects have been achieved. With the advent of new cancer therapies including targeted therapy, immunotherapy, and viral therapy, the survival rate of patients with advanced malignancy is expected to improve. Clinically, much attention should be paid to the reduced incidence of radiation brain necrosis and improved symptoms in patients.

## Figures and Tables

**Table 1:** Recent reports on radiation-induced brain necrosis.

Authors (reference)	Disease	Total case	Year	RT dose (Gy)	Median follow-up time (mo)	RBN (no)	RBN (%)
Huang et al. [6]	NPC	6288	2019	67.4	12.5	24	0.4
Wang et al. [ 7]	NPC	749	2019	66-70.4	48.8	38	5.1
Lu et al. [8]	NPC	4186	2018	68-70	70	188	4.5
Li et al. [9]	NPC	1544	2017	2.0-2.48Gy/28-33 fr	79.7	2	0.13
Shen et al. [10]	NPC	106	2016	66-72	NR	78	73.6
Ilyas et al. [11]	AVM	13941	2018	NR	NR	1844	13.2
Cohen-Inbar et al. [12]	AVM	205	2017	10-50	69	73	35.6
Minniti et al. [13]	MBT	289	2016	3Gy/7 fr or 5Gy/5 fr	10	42	14.5
Swinson and Friedman [14]	MBT	619	2008	10-22.5	12.8	14	2.3
Ruben et al. [15]	Glioma	426	2006	>45 Gy/25 fr	>36	21	4.9

Abbreviations: RT: radiation; NR: not reported; Mo: months; NPC: nasopharyngeal carcinoma; RBN: radiation-induced brain necrosis; AVM: arteriovenous malformations; MBT: metastatic brain tumors.

**Table 2:** Recent reports on treatment for radiation-induced brain necrosis.

Authors (reference)	Year	Treatment	Total case	Median follow-up time (mo)	Resp onse	Efficie nt (%)	Side effects
Xu et al. [87]	2018	Bevacizumab	58	6	38	65.5	Hypertension (20.6%), fatigued (12.1%), infection (6.0%), hemorrhage (6.9%), insomnia (5.2%), headache (5.2%), rash (5.2%), fever (3.4%), blurred vision (1.7%), and hyperglycemia (1.7%)
Xu et al. [87]	2018	Corticosteroid	54	6	17	31.5	Hypertension (18.5%), fatigued (3.7%), infection (11.1%), hemorrhage (3.7%), insomnia (14.8%), headache (7.4%), fever (5.6%), blurred vision (7.4%), hyperglycemia (14.8%), and gain weight (9.3%)
Lam et al. [88]	2012	Steroids, surgery, or observation	174	115	NA	NA	Brain abscess (4%), intracranial hemorrhage (11.6%), and fatal sepsis (27.7%)
Danesh-Meyer et al. [89]	2004	Anticoagulation	1	24	NA	NA	Optic neuropathy
Glantz et al. [90]	1994	Heparin and warfarin	8	17	5	62.5	NA
Williamson et al. [91]	2008	Vitamin E and pentoxifylline	11	8	10	90.1	Nausea and abdominal discomfort
Ohguri et al. [92]	2007	HBO	32	13.7	32	100	Hearing difficulties and ear pain
Cihan et al. [93]	2009	HBO	1	8	NA	NA	NA
Dahl et al. [94]	2019	Bevacizumab	7	4	7	100	NA
Nguyen et al. [95]	2019	Bevacizumab	1	6	NA	NA	Left hypertropia
Carl and Henze [96]	2019	Bevacizumab	58	6	38	65.5	Hypertension (20.6%)
Carl et al. [96]	2019	Corticosteroid	54	6	17	31.5	Hypertension (18.5%)
Aizawa et al. [97]	2018	Surgery	1	43	NA	NA	NA
Li et al. [98]	2018	Bevacizumab	50	6	38	76	NA
Delishaj et al. [99]	2017	Bevacizumab	125	8	114	91.2	Pulmonary embolus (3.2%), hypertension (4.8%), urinary tract infection (0.8%), fatigue (0.8%), proteinuria (0.8%), sagittal sinus thrombosis (0.8%), aspiration pneumonia (0.8%), and pneumonia with severe sepsis (0.8%)
Meng et al. [100]	2017	Bevacizumab	1	2	NA	NA	NA
Wang et al. [5]	2016	NGF	14	36	12	85.7	Pain at the injection site (21.4%)
Chen et al. [ 62]	2019	SFI	48	9	NA	NA	NA
Rao et al. [101]	2014	LITT	15	6	13	75.8	Difficulty walking (6.7%), facial weakness (6.7%), and left sided weakness (6.7%)
Torres-Reveron et al. [102]	2013	LITT	6	6	6	100	None
Fabiano and Alberico [103]	2014	LITT	1	2	NA	NA	NA
Rahmathul et al. [ 104]	2012	LITT	1	2	NA	NA	None

Abbreviations: Mo: months; NPC: nasopharyngeal carcinoma; RBN: radiation-induced brain necrosis; AVM: arteriovenous malformations; MBT: metastatic brain tumors; NA: not applicable; HBO: hyperbaric oxygen; NGF: nerve growth factor; SFI: Shenqi Fuzheng injection; LITT: laser interstitial thermal ablation.

## Additional Points

*Key points* (1) Introduce the incidence of RBN. (2) Analysis of possible molecular mechanisms of RBN. (3) Summarize the existing treatment methods. (4) Proposed prognostic factors and intervention measures for RBN.
